# Affordable Magnetic Hydrogels Prepared from Biocompatible and Biodegradable Sources

**DOI:** 10.3390/polym13111693

**Published:** 2021-05-22

**Authors:** Raluca Ioana Baron, Gabriela Biliuta, Vlad Socoliuc, Sergiu Coseri

**Affiliations:** 1 “Petru Poni” Institute of Macromolecular Chemistry, Romanian Academy, 41 A, Gr. Ghica Voda Alley, 700487 Iasi, Romania; baron.raluca@icmpp.ro; 2 Romanian Academy–Timisoara Branch, Center for Fundamental and Advanced Technical Research, Laboratory of Magnetic Fluids, Mihai Viteazul Ave. 24, 300223 Timisoara, Romania; vsocoliuc@gmail.com; 3 Research Center for Complex Fluids Systems Engineering, Politehnica University of Timisoara, Mihai Vi-teazul Ave. 1, 300222 Timisoara, Romania

**Keywords:** cellulose, PVA, oxidized cellulose, iron oxide magnetic nanoparticles, superparamagnetic hydrogels

## Abstract

Magnetic hydrogels composed of poly(vinyl alcohol) (PVA)/water-soluble tricarboxy cellulose (CO)/magnetic fluids (MFs) have been prepared by a freeze–thaw cycle technique. The system designed here combines the renewability and biocompatibility aspects of PVA and CO, as well as the magnetic properties of MFs, thereby offering special properties to the final product with potential applications in medicine. In the first step, the water-soluble CO is synthesized using a one-shot oxidation procedure and then the aqueous solutions of CO are mixed with PVA solutions and magnetic fluids in the absence of any additional cross-linking agent. The magnetic hydrogels were thoroughly investigated by Fourier transform infrared spectroscopy (FTIR), scanning electron microscopy (SEM), X-ray diffraction (XRD), magnetometry (VSM), and thermogravimetric analysis. The morphological results show an excellent distribution of magnetic particles and CO inside the PVA matrix. The VSM results show that the magnetic hydrogels possess superparamagnetic properties.

## 1. Introduction

Hydrogels are networks of cross-linked polymer chains that are able to retain large solvent quantities without a loss in structural integrity [1]. Hydrogels are extensively used in drug delivery applications [2], agriculture [3], water purification [4,5], tissue engineering [6,7,8,9], soft actuators [10], etc. In the last decade, cellulose has been one of the most preferred compounds, for the hydrogel fabrication due to its natural origin, renewability, compatibility, and abundance. Several hydrogel-based strategies exist: (i) the direct use of native cellulose through physical cross-linking; Despite of these numerous existing methods, there are plenty of obstacles and drawbacks which drastically limit the use of cellulose in magnetic hydrogels production. These limiting factors refer to the use of sophisticated cellulose system solvents, includes the presence of cross-linking chemical agents, and so on; (ii) cellulose derivatives, including methyl cellulose, hydroxypropyl cellulose, hydroxypropylmethyl cellulose, carboxymethyl cellulose, through physical or/and chemical cross-linking; (iii) hybrid cellulose hydrogels, obtained through mixing cellulose with natural polymers, such as chitin, chitosan, starch, alginates, etc., or synthetic polymers, such as poly(vinyl alcohol) (PVA); (iv) cellulose-inorganic hybrid hydrogels obtained through the introduction of inorganic compounds into a hydrogel network, such as biphasic calcium phosphate, charcoal, tungsten carbide, quantum dots, magnetic nanoparticles, etc. For such applications, additional functional groups in the anhydroglucose unit of cellulose are often required, aiming to serve as cross-linking sites, thus the introduction of additional chemical reagents in the system. An oxidation reaction is one of the most common ways to convert cellulose into a value-added derivative and obtain numerous functional groups (–COOH, –CHO). When it is desired to introduce a high content of carboxylic groups in a cellulose structure, a combination of two of the most selective oxidation protocols is employed, as follows: TEMPO is used as a mediator in the selective oxidation of the primary hydroxyl groups (OH-C_6_) in cellulose [11,12,13], and sodium periodate is used as a mediator in the selective oxidation of the secondary hydroxyl groups (OH-C_2_ and OH-C_3_) with the simultaneous removal of C_2_-C_3_ linkage in a one-shot reaction [14]. Due to its water solubility, biocompatibility, and biodegradation, tricarboxy cellulose (CO) exhibits considerable potential uses in different applications, such as forming films, emulsion stabilization, lubrication, and gelling agents. CO may be used in fields ranging from agriculture to food, textile, pharmaceutical, etc. 

On the other hand, PVA is a water-soluble synthetic polymer that is completely biodegradable and widely used in the production of biodegradable hybrid materials with application in various fields, such as packaging [15], water filtration [16], paper [17], and film production [18,19]. From the multitude of hydrogel variants reported in the literature, magnetic hydrogels represent a special category, due to their special properties, namely, their high surface area, good mechanical properties, and tunable porosity and permeability, and magnetic properties. Magnetic hydrogels are made of composite materials that possess biocompatibility, biodegradation, and magnetic responsiveness. The characteristics of magnetic hydrogels depend upon several issues, including the magnetic particles and the component of hydrogels used the magnetic particles and hydrogels’ concentration, and the size and uniformity of magnetic particles within the hydrogels. Magnetic hydrogels, fabricated using iron oxide-based particles and different types of hydrogel matrices, are becoming more and more attractive in biomedical applications by taking advantage of their biocompatibility, controlled architectures, and smart response to magnetic field remotely. Also, magnetic hydrogels have received the most attention for its excellent magnetic and electronic properties. One important property of the magnetic hydrogels is its distinctive form of magnetism, called superparamagnetism. Magnetically responsive hydrogel, as one kind of smart hydrogels, has been introduced into biomedical applications in improving the biological activities of cells, tissues, or organs. This is mainly attributed to its magnetic responsiveness to external magnetic field and obtaining functional structures to remotely regulate physical, biochemical, and mechanical properties of the milieu surrounding the cells, tissues, or organs [20,21,22,23].

In comparison with magnetic hydrogels, various kinds of smart biomaterials (e.g., scaffolds, biofilms, other smart hydrogels), which are activated by external stimuli, such as light, pH, temperature, stress, or charge, have great potential in biomedical applications [24,25,26]. However, the long response time and less precisely controlled architectures of these stimuli-responsive smart biomaterials are the two main limitations. Generally, there are mainly three preparation methods of fabricating magnetic hydrogels: (i) blending method; (ii) in situ precipitation method; (iii) grafting-onto method. In our study, we use an easy method for preparation of magnetic hydrogels–blending method. The blending method has several advantages in the preparation of magnetic hydrogels. Firstly, magnetic particles with homogenous size in hydrogels can be obtained by modifying the stirring speed, the concentration of the substances, and the fabrication period. Secondly, the preparation process is easy to be performed since the preparation and crosslinking of magnetic particles are conducted separately. In our work, we conveniently design a simple procedure for magnetic hydrogel preparation using a renewable water-soluble cellulose derivative, PVA, and a magnetic component, i.e., double-layer oleic acid-coated magnetite (MFs) in the absence of any cross-linking agent. This study aims was to apply a cost-effective technology to prepare magnetic hydrogels by using a simple method of synthesis. The preparation process involved two steps: 1) highly selective oxidation of cellulose to tricarboxy cellulose by using TEMPO/NaBr/NaClO/NaIO_4_ system and 2) synthesis of the magnetic hydrogels through the hydrogen bonding formation between there components. Most importantly, this material was synthesized using biodegradable and biocompatible PVA and CO as raw materials. Concluding we can say that the reported method for the magnetic hydrogel preparation is facile, the components of hydrogels are biodegradable. Also, the water soluble tricarboxy cellulose is a greener, renewable and more sustainable material, and its synthesis is more straightforward, easier to obtain, cost-effective and efficient materials. The prepared hydrogels are characterized using Fourier transform infrared spectroscopy (FTIR), scanning electron microscopy (SEM), X-ray diffraction (XRD), thermogravimetry (TG), and vibrational sample magnetometry (VSM). The various influences of the MFs on the swelling behavior of the prepared hydrogels are also investigated.

## 2. Materials and Methods

### 2.1. Materials

The microcrystalline cellulose type Avicel PH 101 (degree of polymerization of 140) and 99% hydrolyzed poly(vinyl alcohol) (PVA) with average molecular weight of 8.9 × 10^4^–9.8 × 10^4^ were purchased from Sigma-Aldrich. A water based magnetic fluid was prepared at Romanian Academy–Timisoara Branch according to the procedure described in detail in ref. [27]. Magnetite nanoparticles were prepared by chemical coprecipitation of FeSO_4_ and FeCl_3_ iron salts in the presence of NaOH, at 80 °C. The magnetite nanoparticles were sterically stabilized with a hydrophilic double layer of oleic acid (OA) molecules and subsequently dispersed in distilled water. The Fe_3_O_4_/OA.OA water based ferrofluid has Ms = 160 Gs saturation magnetization and ρ = 1.1323 g/cm^3^ density at 26.3 °C. 

### 2.2. Methods

#### 2.2.1. FTIR Analysis

The FTIR spectra were recorded using a Bruker Vertex 70 spectrometer (Bruker, Germany) with a scanning range between 4000 and 400 cm^−1^ using a 2 cm^−1^ resolution and 32 scans. The samples were prepared by the KBr pellet technique (~5 mg sample used in each pellet) before FTIR analysis.

#### 2.2.2. X-ray Diffraction (XRD)

The crystallization patterns of the Avicel, PVA, CO, PVA/CO hydrogel, and magnetic hydrogels were analyzed using a D8 Advance Bruker diffractometer. All analyses were performed in the 2*θ* range between 4° to 40° with a 0.02° s^−1^ data acquisition while employing the reflection method. The current and voltage values were 25 mA and 36 kV, respectively.

#### 2.2.3. Scanning Electron Spectroscopy (SEM) 

The surface morphologies of the studied hydrogels were analyzed using a Quanta 200 scanning electron microscope (FEI Company, Hillsboro, OR, USA) working in a low vacuum mode at 20 kV. The instrument was equipped with an LFD detector and an energy dispersive spectroscopy (EDAX) system for elemental analysis.

#### 2.2.4. Thermogravimetric (TG) Analyses

Thermogravimetric (TG) measurement was performed using a STA 449F1 JUPITER (Netzsch, Germany) device. Temperature and sensitivity calibrations in the temperature range of 30–700 °C were carried out with indium. Approximately 3.0–12 mg of each sample was individually weighed and placed in an alumina pan for TG measurement. The temperature range used was 30 to 700 °C with a heating rate of 10 °C/min under an atmosphere of dry nitrogen at a flow rate of 50 mL/min. The data were processed with the Netzsch Protens 4.2 software package (Selb, Germany).

#### 2.2.5. Magnetic Properties

The magnetization of the magnetic hydrogels was measured using an ADE Technologies VSM880 instrument. The measurements were gathered at room temperature with an applied filed range of +/− 1000 kA/m. The magnetic loading of the hydrogels was determined from static magnetic measurements. For this purpose, the MFs were dried in a vacuum oven at 50 °C in order to obtain water free magnetic nanoparticle (MNP) organosol, i.e., a mixture of MNP and surfactant oleic acid.

#### 2.2.6. Test of Swelling Properties

The freeze-dried magnetic hydrogels were immersed in deionized water at room temperature in order to determine the swelling properties. At certain intervals, the samples were taken out of the water, the excess liquid was removed easily and quickly with the help of filter paper, and they were weighed after the gravimetric swelling process.

The swelling ratio was calculated by Equation (1):

(1) $$S\left(\%\right)=\frac{W_{t}-W_{d}}{W_{d}}\times 100$$

*W_t_*—shows the mass of the hydrogel in the swollen state at time *t*, *W_d_*—shows the mass of the hydrogel in the dry state. For each hydrogel, three experiments were performed in parallel, the final value being their average. All the swelling experiments were done in triplicate, and the mean values were used to represent the swelling behavior of each sample.

#### 2.2.7. Hydrogels Stability Studies

The stability of the as prepared hydrogels was studied as follows: a piece of each sample (100 mg) was immersed in 10 mL solutions, with different pH values: pH = 4 (Merck KGaA buffer solution citric acid/sodium hydroxide/hydrogen chloride), pH = 5.6 (distilled water), pH = 7 (Merck KGaA buffer solution potassium dihydrogen phosphate/di-sodium hydrogen phosphate) and pH = 9 (Merck KGaA buffer solution boric acid/potassium chloride/sodium hydroxide). All solutions containing the hydrogel samples were heated at 60 °C and maintain at this temperature for 2 h. Further, the solutions were cooled on an ice bath, and the samples were removed allowing drying for 2 h at ambient conditions, then dried in an oven at 60 °C for 2 h. Finally, the samples were weighed. The whole procedure was repeated 5 times. Independently, one piece of each sample has been subjected to the ultrasonic treatment. The sample was immersed in distilled water until it reached swelling equilibrium, then weighed, and placed in 100 ml of water, being subjected to ultrasound treatment for 10 min, using an Ultrasonic Processor GEX-500 sonicator, operating at 500 Watts and 20 kHz frequency, on an ice bath to prevent overheating.

### 2.3. Cellulose Oxidation

Tricarboxy cellulose was prepared via one-shot step oxidation according to our reported method [14]. TEMPO (2.5 mmol), sodium periodate (12.5 mmol), and NaBr (40 mmol) were dissolved in distilled water under vigorous stirring and then 5 g of microcrystalline cellulose was suspended in the reaction mixture. A 9% NaClO solution (40 mmol) was added to the cellulose slurry under continuous stirring and the resulting suspension was stirred for 6 h at room temperature and in a dark condition. The pH of the suspension was carefully maintained at about 10.5 by adding a 2 M NaOH solution. The oxidation reaction was stopped with ethanol and the oxidized cellulose was filtered and washed several times with deionized water and a 0.5 M HCl solution. The water-soluble fraction was precipitated with ethanol, where the formed precipitate was collected by centrifugation and then purified via dialysis and recovered by freeze-drying.

### 2.4. Fabrication of PVA/CO/MFs Hydrogels

A PVA solution (solution concentration of 5% *w*/*w*) was prepared in Millipore water and was left to stir overnight at 80 °C to ensure complete dissolution of the polymer. Tricarboxy cellulose (CO) (solution concentration of 5% *w*/*w*) was dissolved in Millipore water by magnetic stirring at room temperature. The MFs was dispersed by sonication for 15 min before use. Thus, four hydrogels were prepared by adding increasing amounts of the MFs to the PVA/CO solutions in the following ratios: 9/1/0, 9/1/0.005, 9/1/0.05, and 9/1/0.125 (*w*/*w*), where the samples are labeled as PVA/CO, PVA/CO/MFs-1, PVA/CO/MFs-10, and PVA/CO/MFs-25, respectively. After mixing, the solutions experienced 3 freeze–thaw cycles in order to obtain physical networks. The resulted hydrogels were dried by lyophilization and investigated by different methods.

## 3. Results and Discussion

**Scheme 1:**
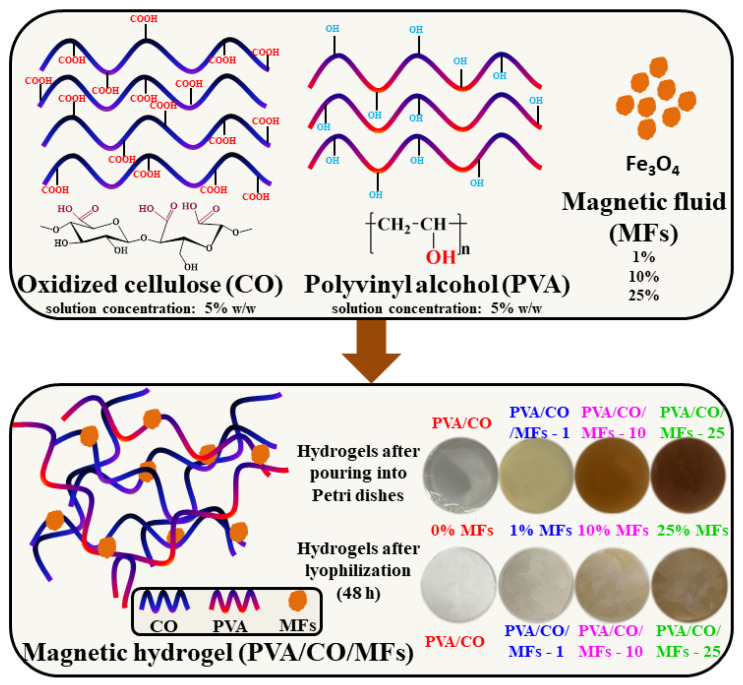
The illustrative representation of PVA/CO/MFs magnetic hydrogel preparation, emphasizing the color changes that occurred after the addition of different MFs amounts.

The preparation of the PVA/CO/MFs magnetic hydrogels is depicted in Scheme 1. CO, having three carboxyl groups, could easily form physical networks with PVA. The Fe_3_O_4_ nanoparticles were stabilized and incorporated homogeneously into the resulting network. 

The preparation process involved two steps. In the first step, tricarboxy cellulose was synthesized by employing a double-oxidizing system while combining the TEMPO/NaBr/NaOCl protocol with NaIO_4_ at room temperature and a pH of 10. In the second step, the magnetic hydrogels were obtained through the inclusion of MFs into the complex hydrogen bond network that resulted from the interaction between the carboxylic groups of CO and the hydroxyl groups of PVA.

### 3.1. FTIR Analysis

**Figure 1:**
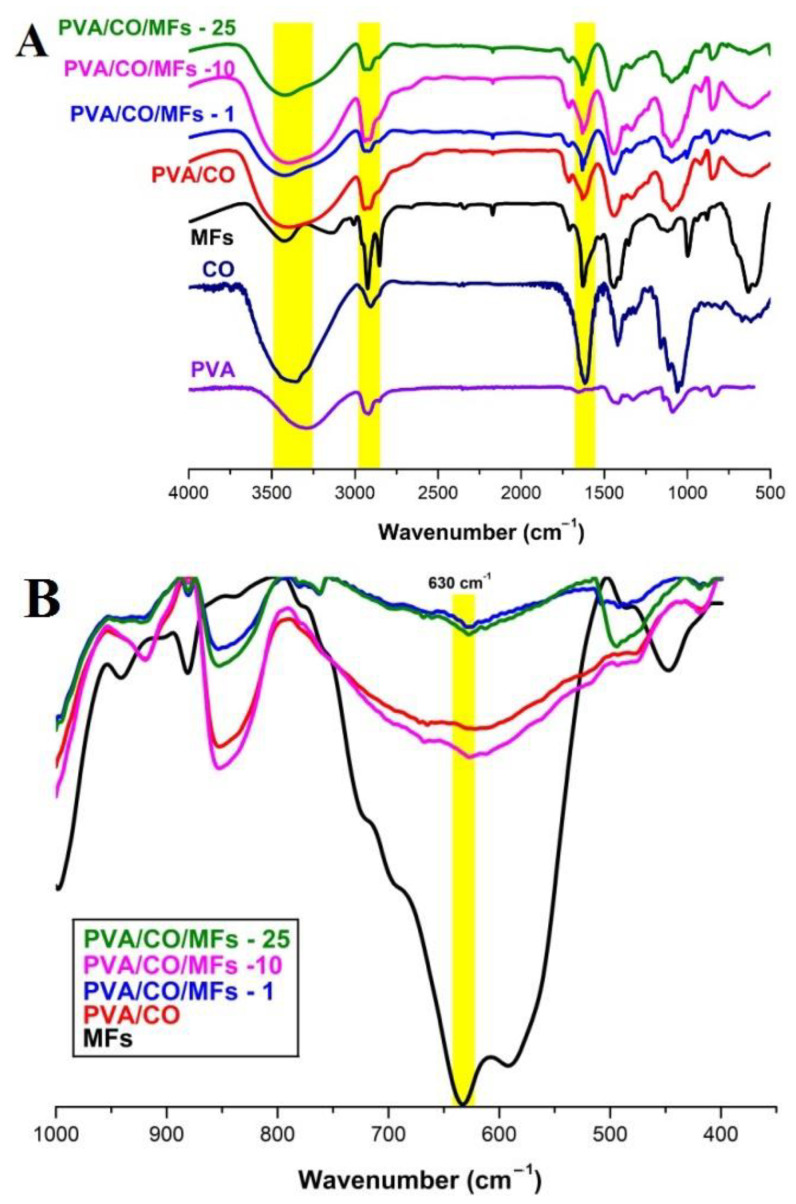
FTIR spectra of CO, PVA, MFs, PVA/CO, PVA/CO/MFs-1, PVA/CO/MFs-10, PVA/CO/MFs-25 (**A**), and PVA/CO/MFs-25, PVA/CO/MFs-10, PVA/CO/MFs-1, PVA/CO, MFs (400–1000 cm^−1^) (**B**).

The chemical structures of the magnetic hydrogels were characterized by Fourier transform infrared spectroscopy. Figure 1 shows the spectra of the MFs, PVA/CO, and magnetic hydrogels with 1%, 10%, and 25% MFs, respectively. For the MFs, the characteristics peaks at 3426 and 1627 cm^−1^ were due to the O-H stretching vibration adsorption and H-O-H in-plane bending vibration, and the peak at 630 cm^−1^ was assigned to the Fe-O stretching. In the case of PVA/CO, a broad and intense peak at 3438 cm^−1^ was ascribed to the stretching vibration of OH. The peaks at 2981, 1442, and 1136 cm^−1^ were associated with the vibration of C-H and C-O-C stretching and C-H bending, respectively. The peak at 1710 cm^−1^ was assigned to the stretching vibration of –COO groups in their acidic form. After the incorporation of MFs, the peaks corresponding to OH groups became broader and shifted to lower wavenumbers when compared with PVA/CO, suggesting the formation of new hydrogen bonds and interaction among these components. Compared with the initial two components (PVA and CO), all the magnetic hydrogels exhibited a new peak at 630 cm^−1^, indicating the successful incorporation of MFs.

### 3.2. X-Ray Diffraction (XRD)

**Figure 2:**
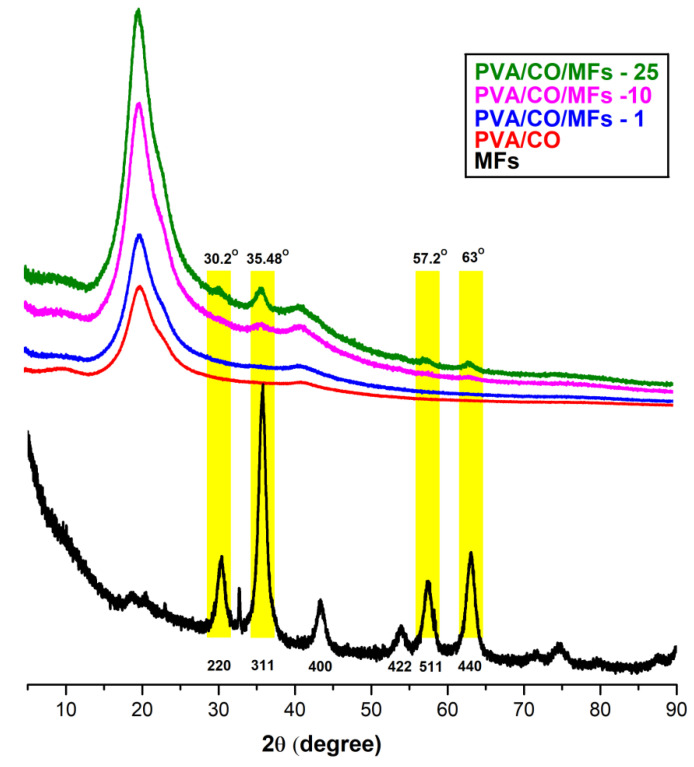
The XRD spectra of MFs, PVA/CO, and the magnetic hydrogels PVA/CO/MFs-1, PVA/CO/MFs-10, and PVA/CO/MFs-25.

The X-ray diffraction patterns of PVA/CO/MF magnetic hydrogels are presented in Figure 2, as well as the PVA/CO and MF precursors. The neat PVA/CO hydrogel exhibited a major peak around *2θ* = 19.8° corresponding to semi-crystalline PVA contribution, and a shoulder peak at *2θ* = 22.8° corresponding to oxidized cellulose, which could be ascribed as a “cellulose fingerprint”.

According to Powder Diffraction FileTM (PDF^®^), all the diffraction peaks of the MFs for the face center cubic structure of magnetite could be indexed as follows: (220), (311), (400) (422), (511), and (440). Comparing the diffractograms for the neat hydrogel and magnetic hydrogels, it can be observed that higher magnetic loading induces the presence of peaks associated with the crystal planes of MFs at *2θ* = 30.2°, 35.48°, 57.2°, and 63°, especially for the PVA/CO/MFs-10 and PVA/CO/MFs-25 samples. Moreover, X-ray diffractograms of Avicel, CO, PVA/CO and PVA samples are comparatively presented in Figure S1, see Supporting Information section. PVA show a unique crystalline contribution peak, corresponding to the (1 0 1) semicrystalline plan around *2θ* = 19.8°. Avicel and CO samples are almost identical, with the two main diffraction regions characteristic for cellulose I: one attributed to the amorphous area, with peaks at *2θ* = 14.8° and 16.6°, and another one for the crystalline zone, with the diffraction pattern at *2θ* = 22.8°. Interestingly, the peak around *2θ* = 40° corresponding to the (400) crystal plane, present in magnetic hydrogels spectra is shifted in the hydrogel samples, (*2θ* = 43.1°) due to additional interactions and/or reorganization of the hydrogen bonding networks between components.

### 3.3. Morphology of Magnetic Hydrogels

**Figure 3:**
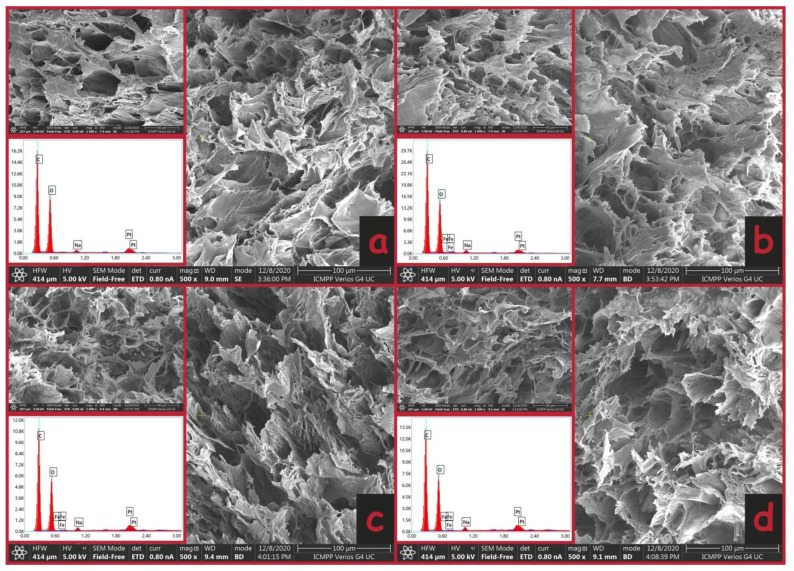
The SEM images and EDX spectra of the prepared hydrogels PVA/CO (**a**), PVA/CO/MFs-1 (**b**), PVA/CO/MFs-10 (**c**) and PVA/CO/MFs-25 (**d**).

The morphological characteristics of the magnetic hydrogels were determined by SEM. As seen in the SEM images provided in Figure 3, the magnetic hydrogels exhibited a porous surface without obvious particle distribution, indicating uniform dispersion among these components. It was observed that the pore sizes of the magnetic hydrogels did not differ in essence from the pore size of an unloaded hydrogel. The presence of MFs in the composition of hydrogels was also confirmed by EDX, which shows the existence of iron (Fe) and oxygen (O) elements (see the inserts in Figure 3) in the structure of hydrogels.

### 3.4. Thermogravimetric Analysis (TG) 

**Figure 4:**
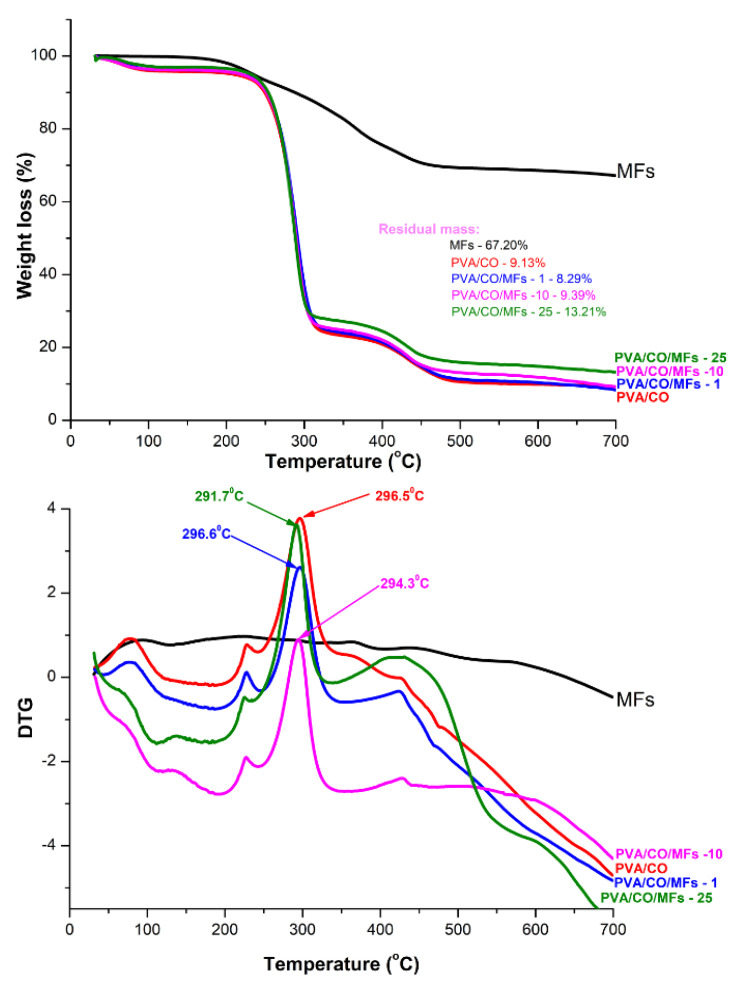
TGA and DTG curves of MFs and magnetic hydrogels.

Figure 4 shows the TG curves of magnetic hydrogels and MFs in a nitrogen atmosphere. Based on the TG curves, the thermal degradation of the magnetic hydrogels could be divided into three stages. The first stage, which appeared at 50–120 °C, corresponds to the evaporation of water. The second stage, featuring major weight loss, appeared at 220–320 °C and corresponds to the structural degradation of PVA and CO. The third weight loss occurred at 320–500 °C and corresponds to the maximum degradation temperature of the hydrogels until charring. Comparing the PVA/CO hydrogel, for the magnetic PVA/CO/MFs hydrogels, the initial decomposition temperatures were similar, indicating that the introduction of MFs had no obvious influence on the decomposition temperature. The DTG peak of PVA/CO (296 °C) was almost similar to the DTG peaks of the magnetic hydrogels (296 °C for PVA/CO/MFs-1, 294 °C for PVA/CO/MFs-10, and 291 °C for PVA/CO/MFs-25), indicating that introduction of MFs has not accelerated the decomposition of the hydrogels. The residual mass of the magnetic hydrogels was slightly higher than PVA/CO hydrogel due to the presence of the MFs in the structure of the hydrogel (Table 1).

### 3.5. Hydrogels Stability under Various Environments

The thermal/hydrolytic stability of newly synthesized materials, either in the form of films, fibers or hydrogels, represents a crucial parameter, especially when these materials are intended to be applied in fields such as: medical, biomedical or food. There are several testing protocols proposed in the literature [28]. To check the hydrogels’ stability at different pH values and under repetitive heating–cooling cycles, we have done some preliminary tests, measuring the samples’ weights before and after each heating–cooling cycle of the hydrogels immersed in four solution at different pH values (4, 5.6, 7 and 9). After performing the experiments, we are able to confirm that all the samples displayed good hydrolytic stability, the investigated samples did not show weight loss after seven heating–cooling cycles in solutions with different pH values. Moreover, the ultrasound treatment of each sample was done at ultrasonic amplitude of 21 μm for 10 min. Then, the sample was weighed, noting that no mass loss occurred. However, when the ultrasonic amplitude was increased to 25 μm, the sample was completely disintegrated.

### 3.6. Magnetic Properties (VSM) of the as Prepared Magnetic Hydrogels

**Figure 5:**
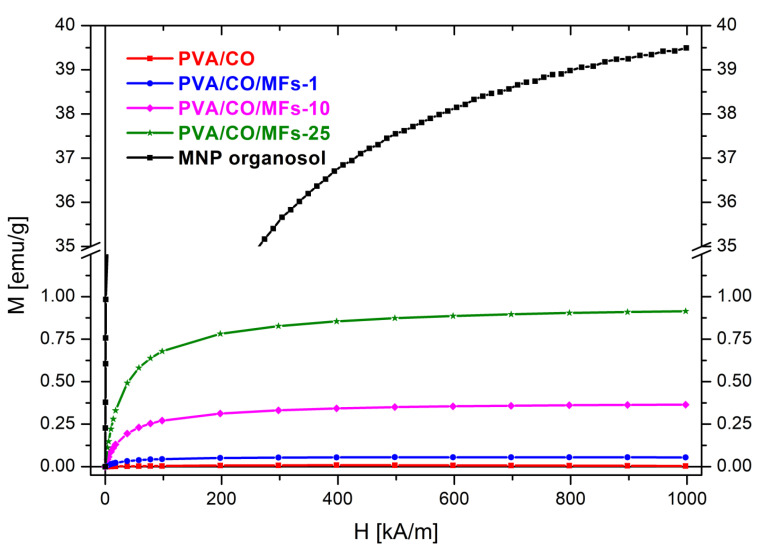
Magnetization curves of the hydrogels and MNP organsol.

In Figure 5 are presented the magnetization curves of the hydrogels and MNP organsol. The MNP organosol and the magnetic hydrogels (PVA/CO/MFs-1, PVA/CO/MFs-10 and PVA/CO/MFs-25) show superparamagnetic behaviour. The magnetic hysteresis measurements showed no magnetic coercivity and remanence. The reference hydrogel PVA/CO has zero magnetization.

Table 2 provides the magnetometry samples’ masses and saturation (H = 1000 kA/m) values of magnetization (M_sat_) and magnetic moment (μ_sat_). Using the masses and the saturation magnetic moment, the MNP organosol load of PVA/CO/MFs-1, PVA/CO/MFs-10 and PVA/CO/MFs-25 was calculated as weigh percentage relative to the polymer (w% polymers).

**Figure 6:**
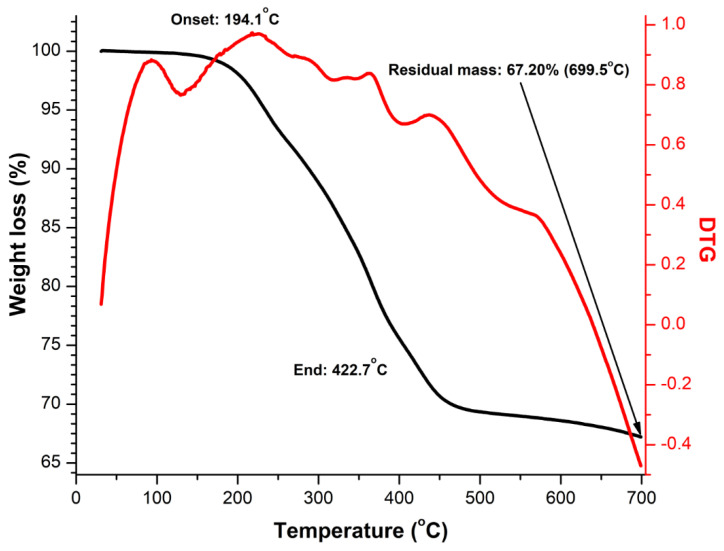
TGA measurement of the MNP organosol.

In order to accurately assess the MNP loading in the prepared hydrogels, TGA and DTG curve of neat MFs in a nitrogen atmosphere was recorded, Figure 6. It can be seen that the thermal degradation curve of MFs shows no significant initial water loss, the most important degradation step observed is in the temperature range 150–400 °C related with the decomposition of the organic surfactant, the residual mass being 67.20 w%, value which allows us to calculate the MNP load of the hydrogels: 0.09%, 0.64% and 1.36% respectively (Table 2). From the process of the MFs drying, the MNP organosol weight percentage in the ferrofluid was found to be 16.2 w% MFs. With this, using the hydrogels’ MNP organosol load, we calculated the effective ferrofluid weight percentage (final MFs load) in the final products of the synthesis processes PVA/CO/MFs-1, PVA/CO/MFs-10, and PVA/CO/MFs-25: 0.85%, 6.05% and 16.70% respectively (Table 2). The comparison of these values with the initial design values (MFs load) shows that MNP are lost during the synthesis process.

### 3.7. Swelling Properties of Magnetic Hydrogels

**Figure 7:**
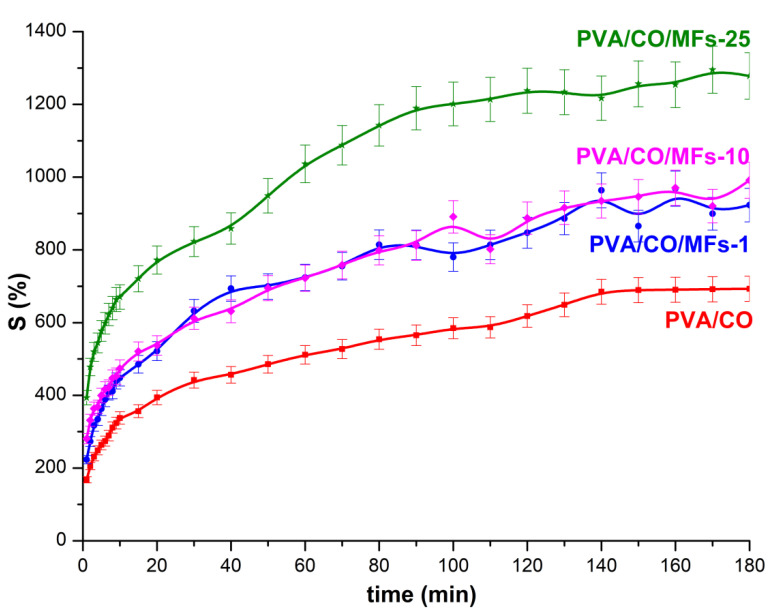
Degree of swelling of hydrogels in water.

The swelling properties, are crucial parameters when study new hydrogels, and usually use degree of swelling to define hydrogels. These properties depend on several factors such as network density, solvent nature, polymer solvent interaction parameter. The results of the swelling ratio test for PVA/CO/MFs hydrogels in comparison with those for PVA/CO hydrogel in deionized water are shown in Figure 7.

It could be seen from the figure that the swelling degree of all PVA/CO/MFs hydrogels was higher than that of PVA/CO hydrogel, as a first indication that a stable network was formed after MFs embedded. Also, the capacity of the water absorption was increase with the increased of the MFs used. The swelling ratios were slightly increased in time, which means that the network structure of the PVA/CO/MFs hydrogels was more stable, observation which is consistent with SEM images, revealing a homogeneously and unitary morphology. The high amounts of hydroxyl groups originate from PVA, the carboxyl groups from oxidized cellulose and also the concentration of MFs, have a great impact on the swelling behavior of the prepared hydrogels. The highly hydrophilic feature of the magnetic hydrogels allows the water to easily penetrate the pores of the hydrogels [29]. The swelling values increased, as the amount of MFs in the hydrogel increased. However, there are no significant differences when 1% or 10% MFs were added in hydrogels preparation, even if the MFs content is higher, it does not induce a dramatic change in behavior towards the hydrogel by 1% MFs, thus suggesting that the amount added is still insufficient to affect the swelling properties, the noticeable changes of these properties being observed at higher MFs concentrations, i.e., 25%. The addition of small quantities of fillers, i.e., magnetic components, carbon nanotubes, graphene, will drastically change the hydrogel properties: swelling, thermal, mechanical, etc. But these properties are not necessarily linearly linked with the total amount of the added filler. There is a critical concentration of the additive, up to which some properties do not undergo significant changes, but after this concentration, those properties are significantly affected. Particularly, the swelling behavior of a certain hydrogel is not a simple process, which do not follow a linear trend. There are several factors affecting the swelling behavior, such as: pH, temperature, the hydrogel composition, etc., all of them dictating eventually an inhomogeneous or homogeneous swelling behavior. To additionally characterize hydrogels, an important parameter is the water diffusion, determined by combining inflation experiments with different mathematical models. The study of water diffusion phenomena in superabsorbent hydrogels is of great interest because it clarifies the behavior of the polymer for applications in biomedicine, pharmaceutical or tissue engineering. The diffusion of water in crosslinked hydrogels involves the migration of water molecules in certain spaces of the hydrogel network. In order to study the diffusion of water through the pores of hydrogels, the data obtained (up to 60% of the maximum swelling capacity) were used, which were processed according to Fick’s law, expressed by the Equation 2:

(2) $$\frac{M_{t}}{M_{eq}}=Kt^{n}$$

wherein, *M_t_* and *M_eq_* represent the amount of solvent absorbed by the hydrogel at time *t*, respectively at equilibrium, *K* is the swelling constant characteristic of the system, and *n* represents the diffusion coefficient depending on the nature of the solvent transport process [30]. The constants *n* and *K* were calculated from the slope and intercepts of the plots of *ln(M_t_/M_eq_)* versus *ln(t)* from the experimental data, graphs shown in Figure S2. Consistent with these graphs, it can be seen that the swelling data fit the Korsmeyer-Peppas model accordingly. According to the classification of diffusion models, there are several cases: *n* < 0.5 is dominated by *Fickian diffusion*, where water transport is directed by a spontaneous concentration gradient; 0.5 < *n* < 1 is dominant *Fickian diffusion (anomalous)*-water absorption due to both the process of water diffusion and the relaxation of polymer chains; *n* = 1-the transport is driven mainly by the macromolecular relaxation of the polymer chains; *n* > 1-macromolecular relaxation and erosion of polymer chains together contribute to water diffusion. Thus, in our case, due to the fact that the diffusion coefficient, *n*, is between 0.2898 and 0.2095 (as shown in the Table 3), it can be said that the kinetic mechanism is predominant by Fickian diffusion and is achieved mainly by diffusion.

## 4. Conclusion

Magnetic hydrogels, fabricated using iron oxide-based particles stabilized and dispersed by different types of hydrogel matrices, are becoming more and more attractive in biomedical applications, in improving the biological activities of cells, tissues, or organs, by taking advantage of their biocompatibility, controlled architectures, and smart response to magnetic field remotely. They received a great deal of attention because of the excellent magnetic and electronic properties. One important property of the magnetic hydrogels is its distinctive form of magnetism, called superparamagnetisms. In this paper, we report an efficient and simple one-step process for the preparation of the superparamagnetic PVA/CO/MFs hydrogels. The method is extremely simple and cost-effective which may lead to the development of magnetic hydrogels with multiple applications in the areas of biotechnology/biomedicine. Most importantly, these magnetic hydrogels were prepared using two biodegradable and biocompatible materials. However, there are also some limitations that could be addressed in the future, including the asymmetric distribution of magnetic particles within the hydrogels and the diffusion of magnetic particles when the magnetic hydrogels were submerged in a solution.

## Figures and Tables

**Table 1:** TGA and DTG results for the magnetic hydrogels.

Sample	DTG Peak (°C)	Residual Mass (%)
PVA/CO	296.5	9.13
PVA/CO/MFs-1	296.6	8.29
PVA/CO/MFs-10	294.3	9.39
PVA/CO/MFs-25	291.7	13.21
MFs		67.20

**Table 2:** Saturation magnetization data and magnetic loading calculations.

Magnetic Hydrogels	Initial MF Load [wt %]	Sample Mass [g]	M_sat_ [emu/g]	μ_sat_ [memu]	MNP Organosol Load [wt %]	MNP Load [wt %]	Final MF Load [wt %]
PVA/CO/MFs-1	1	0.0064	0.0539	0.345	0.14	0.09	0.85
PVA/CO/MFs-10	10	0.0068	0.3646	2.479	0.93	0.64	6.05
PVA/CO/MFs-25	25	0.0079	0.9147	7.226	2.37	1.36	16.70
MNP Organosol	-	0.1175	39.4939	4633.740	-	-	-

**Table 3:** Kinetic parameters of swelling.

Hydrogels	*R* ^2^	*k*	*n*	Swelling Mechanism
PVA/CO	0.9962	0.1736	0.2649	Fickian diffusion
PVA/CO/MFs-1	0.9951	0.1780	0.2898	Fickian diffusion
PVA/CO/MFs-10	0.9951	0.2231	0.2293	Fickian diffusion
PVA/CO/MFs-25	0.9934	0.2758	0.2095	Fickian diffusion

## Data Availability

The data presented in this study are available on request from the corresponding authors.

## Supplementary Materials

The following are available online at https://www.mdpi.com/article/10.3390/polym13111693/s1, Figure S1. The XRD spectra of cellulose (Avicel), oxidized cellulose (CO), PVA, and the hybrid hydrogel sample (PVA/CO), Figure S2. Swelling kinetic plots (ln (Mt/Meq) versus ln (t)) of the hydrogels.
