# Supplementary material for: Affordable Magnetic Hydrogels Prepared from Biocompatible and Biodegradable Sources
Source: Polymers (Basel). 2021 May 22;13(11):1693. doi: 10.3390/polym13111693 (PMC8196864; doi:10.3390/polym13111693)
Supplement: Supplementary file 1 [file polymers-13-01693-s001.zip › polymers-1202821-supplementary.pdf]

polymers

Article

Affordable Magnetic Hydrogels Prepared from Biocompatible and Biodegradable Sources

Raluca Ioana Baron [1], Gabriela Biliuta [1,*], Vlad Socoliuc [2,3] and Sergiu Coseri [1,*]

[1] "Petru Poni" Institute of Macromolecular Chemistry, Romanian Academy, 41 A, Gr. Ghica Voda Alley, 700487 Iasi, Romania; baron.raluca@icmpp.ro
[2] Romanian Academy – Timisoara Branch, Center for Fundamental and Advanced Technical Research, La-boratory of Magnetic Fluids, Mihai Viteazul Ave. 24, 300223 Timisoara, Romania; vsocoliuc@gmail.com
[3] Politehnica University of Timisoara, Research Center for Complex Fluids Systems Engineering, Mihai Vi-teazul Ave. 1, 300222 Timisoara, Romania
* Correspondence: biliuta.gabriela@icmpp.ro (G.B.); coseris@icmpp.ro (S.C.)

Citation: Baron, R.I.; Biliuta, G.; Socoliuc, V.; Coseri, S. Affordable Magnetic Hydrogels Prepared from Biocompatible and Biodegradable Sources. *Polymers* **2021**, *13*, 1693. https://doi.org/10.3390/polym13111693

Academic Editor: Anton Blencowe

Received: 14 April 2021
Accepted: 13 May 2021
Published: date

Publisher's Note: MDPI stays neutral with regard to jurisdictional claims in published maps and institutional affiliations.

Copyright: © 2021 by the authors. Submitted for possible open access publication under the terms and conditions of the Creative Commons Attribution (CC BY) license (http://creativecommons.org/licenses/by/4.0/).

Supplementary Infirmation

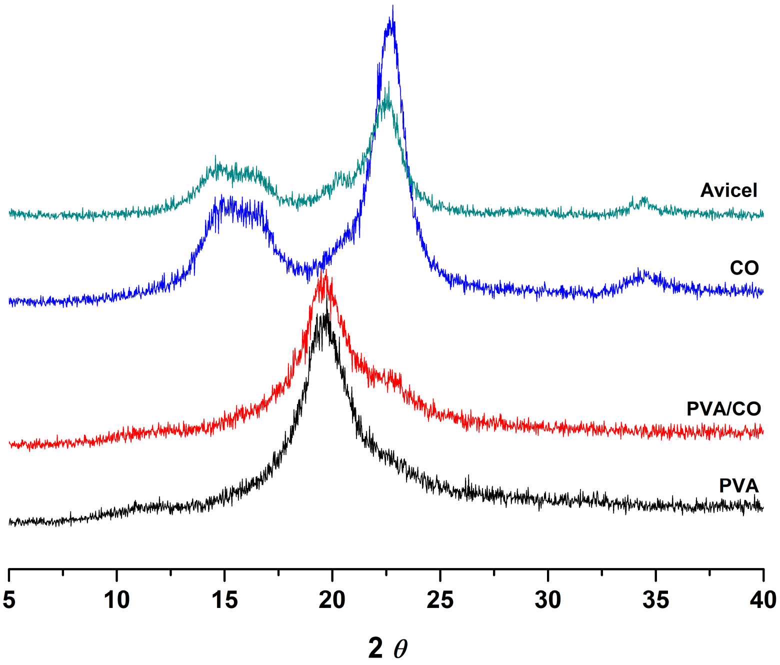

Figure S1. The XRD spectra of cellulose (Avicel), oxidized cellulose (CO), PVA, and the hybrid hydrogel sample (PVA/CO).

* All these analyses were performed in the 2θ range between 4° to 40°, with a 0.02° s^{-1} data acquisition, employing the reflection method.

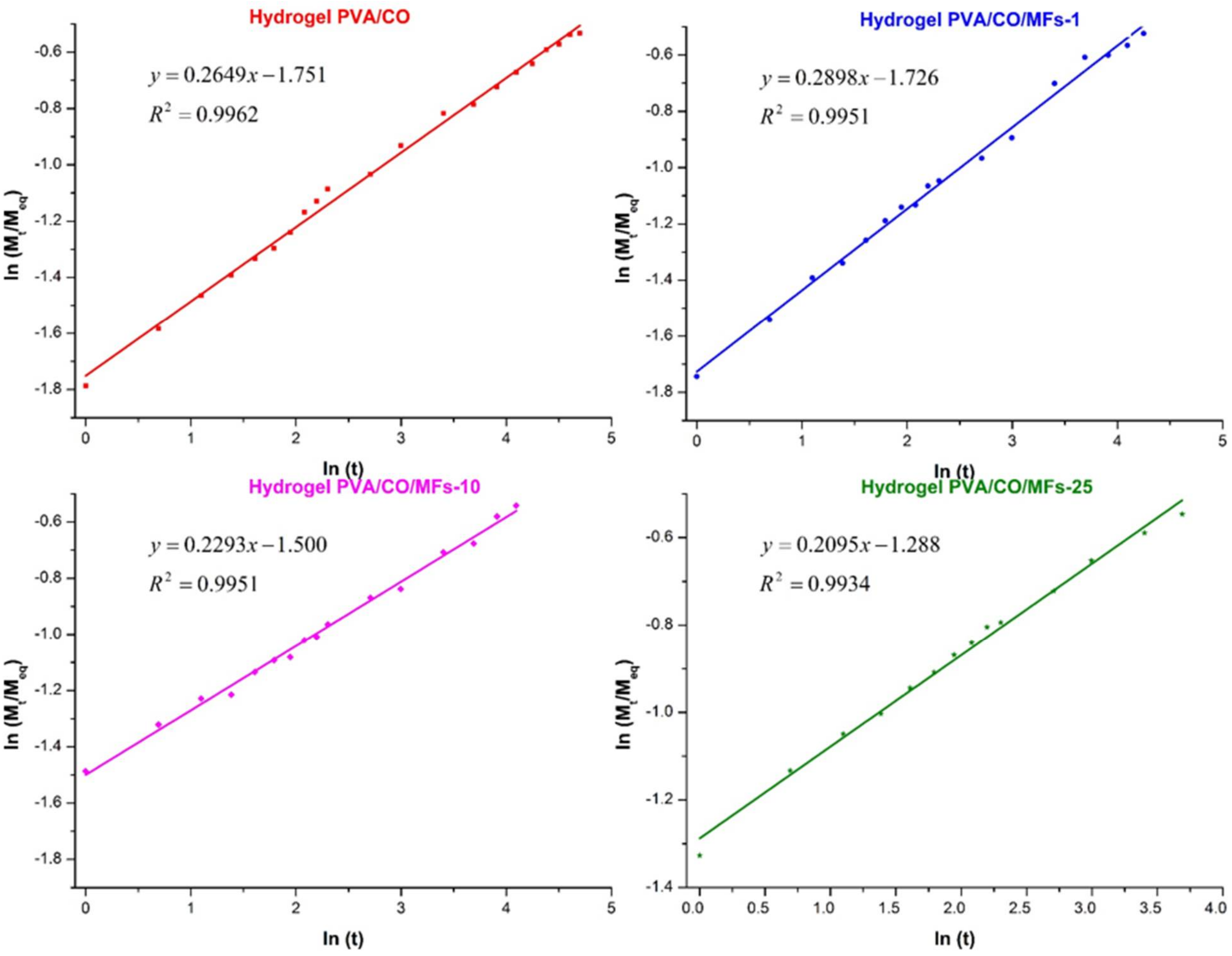

Figure S2. Swelling kinetic plots (ln (M_t/M_{eq}) versus ln (t)) of the hydrogels.
